# Supplementary material for: Concentration-Dependent Attenuation of Pro-Fibrotic Responses after Cannabigerol Exposure in Primary Rat Hepatocytes Cultured in Palmitate and Fructose Media
Source: Cells. 2023 Sep 9;12(18):2243. doi: 10.3390/cells12182243 (PMC10526512; doi:10.3390/cells12182243)
Supplement: Supplementary file 1 [file cells-12-02243-s001.zip › cells-2527349-supplementary.pdf]

1.1. Concentration-dependent influence of cannabigerol on transforming growth factor beta 1 changes in post-incubation media and primary rat hepatocytes exposed to palmitate in combination with fructose

An increment in the protein expression of TGF-β1 was detected in both PA-F groups (+64.9% and +67.2%, $p<0.05$, Figure S1A and S1B, respectively). The TGF-β1 protein expression was increased after hepatocytes treatment with 5μM CBG and 10μM CBG alone or in combination with PA-F exposure (+33.7%, +33.2%, +82.2% and +5.5%, $p<0.05$, Figure S1A, respectively) compared to the Control group. In relation to the PA-F group, the protein expression of TGF-β1 was reduced in lysates of hepatocytes exposed to PA-F and 10μM CBG (-36.0%, $p<0.05$, Figure S1A). In the protein expression of TGF-β1, we also noticed a decrease after hepatocytes treatment with 15μM CBG alone (-32.0%, $p<0.05$, Figure S1B) and an increase after hepatocytes treatment with 15μM CBG in the PA-F condition (+70.3%, $p<0.05$, Figure S1B) in relation to the Control group. In addition, the TGF-β1 protein expression was enhanced in the PA-F + 25μM CBG group (+160.4% and +55.8%, $p<0.05$, Figure S1B) and lowered in the PA-F + 30μM CBG group (-13.4% and -48.2%, $p<0.05$, Figure S1B) than in the Control and PA-F groups, respectively.

An increment in the mRNA expression of TGF-β1 was detected in both PA-F (+19.3%, and +26.7%, $p<0.05$, Figure S1C and S1D, respectively). In the PA-F condition, the mRNA expression of TGF-β1 was augmented by 5μM CBG treatment (+7.2%, $p<0.05$, Figure S1C, vs. Control group). In the PA-F + 10μM CBG group, we observed an attenuation in the mRNA expression of TGF-β1 (-14.1%, $p<0.05$, Figure S1C) in relation with the PA-F group. The mRNA expression of TGF-β1 was also significantly changed in experimental group treated with palmitate and fructose with the addition of cannabigerol (PA-F + 15μM CBG: +43.1% and +12.9%, PA-F + 25μM CBG: +60.3% and +26.5%, PA-F + 30μM CBG: -17.6% and -35.0%, $p<0.05$, Figure S1D) compared to the Control and PA-F groups, respectively.

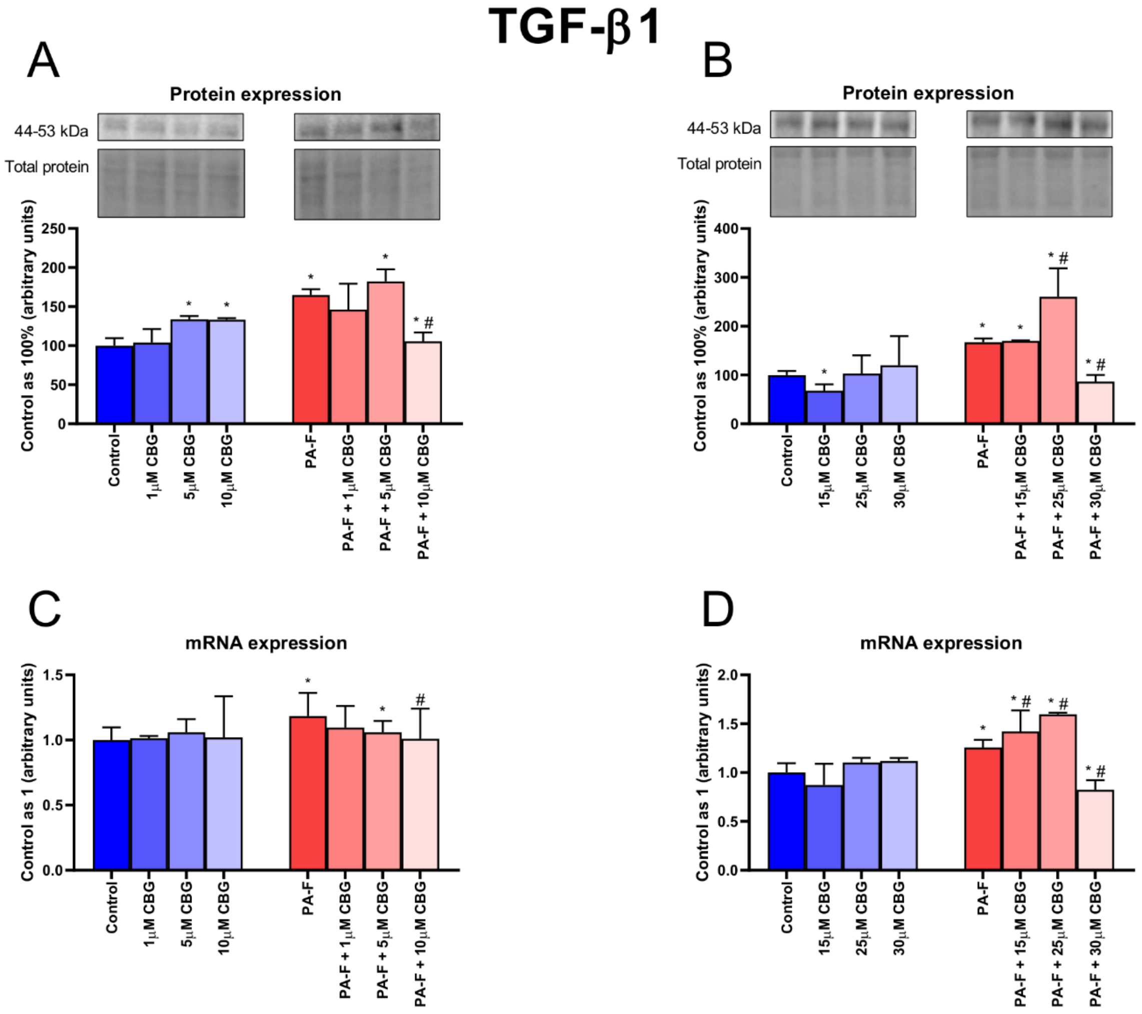

Figure S1. The influence of cannabigerol (CBG), in a concentration-dependent manner, on the transforming growth factor beta 1 (TGF-β1) protein expressions (A, B), mRNA expressions (C,D) in hepatocytes exposed to standard media (Control) or palmitate and fructose media (PA-F). The cells were treated with different concentrations of CBG for 48h. The TGF-β1 protein and mRNA expression assays were performed by the Western blot and RT-PCR techniques, respectively. The data were expressed as mean ± SD of six independent determinations and presented as a percentage of TGF-β1 changes relative to the Control (set as 100% for Western blot and as 1 for RT-PCR). The significant differences were indicated in comparison with the Control group (*$p<0.05$) and PA-F group ($^{\#}p<0.05$).

1.2. Concentration-dependent influence of cannabigerol on matrix metalloproteinase 2 changes in post-incubation media and primary rat hepatocytes exposed to palmitate in combination with fructose

The hepatocytes treatment with 5μM CBG and 10μM CBG in combination with the palmitate and fructose caused a raise in the MMP-2 protein expression (PA-F + 5μM CBG: +92.4% and +74.0%, PA-F + 10μM CBG: +70.2% and +53.9%, $p<0.05$, Figure S2A) compared to the Control and PA-F groups, respectively. In the protein expression of MMP-2, we also observed a decrease in the following examined groups: 25μM CBG, 30μM CBG, PA-F + 15μM CBG, PA-F + 25μM CBG, PA-F + 30μM CBG (-25.5%, -27.0%, -34.7%, -40.0%, -39.5%, $p<0.05$, Figure S2B, respectively) in relation to the Control group. Moreover, the hepatocytes treatment with high concentration of CBG in the PA-F condition induced a diminishment in the protein expression of MMP-2 (PA-F + 15μM CBG: -38.7%, PA-F + 25μM CBG: -43.4%, PA-F + 25μM CBG: -43.0%, $p<0.05$, Figure S2B) than in the PA-F group.

In both PA-F groups, we observed a decrease in the mRNA expression of MMP-2 (-50.0% and -37.0%, p<0.05, Figure S2E and S2F, respectively). The mRNA expression of MMP-2 was also significantly reduced in selected examined groups (PA-F + 5μM CBG: -26.3%, PA-F + 10μM CBG: -45.8%, PA-F +

15μM CBG: -51.9%, PA-F + 30μM CBG: -55.6%, $p<0.05$, Figure S2C and S2D) compared to the proper Control group. We also noticed a decline in the MMP-2 mRNA expression in the hepatocytes treatment with 30μM CBG in the PA-F condition (-29.4%, $p<0.05$, Figure S2D, vs. PA-F group).

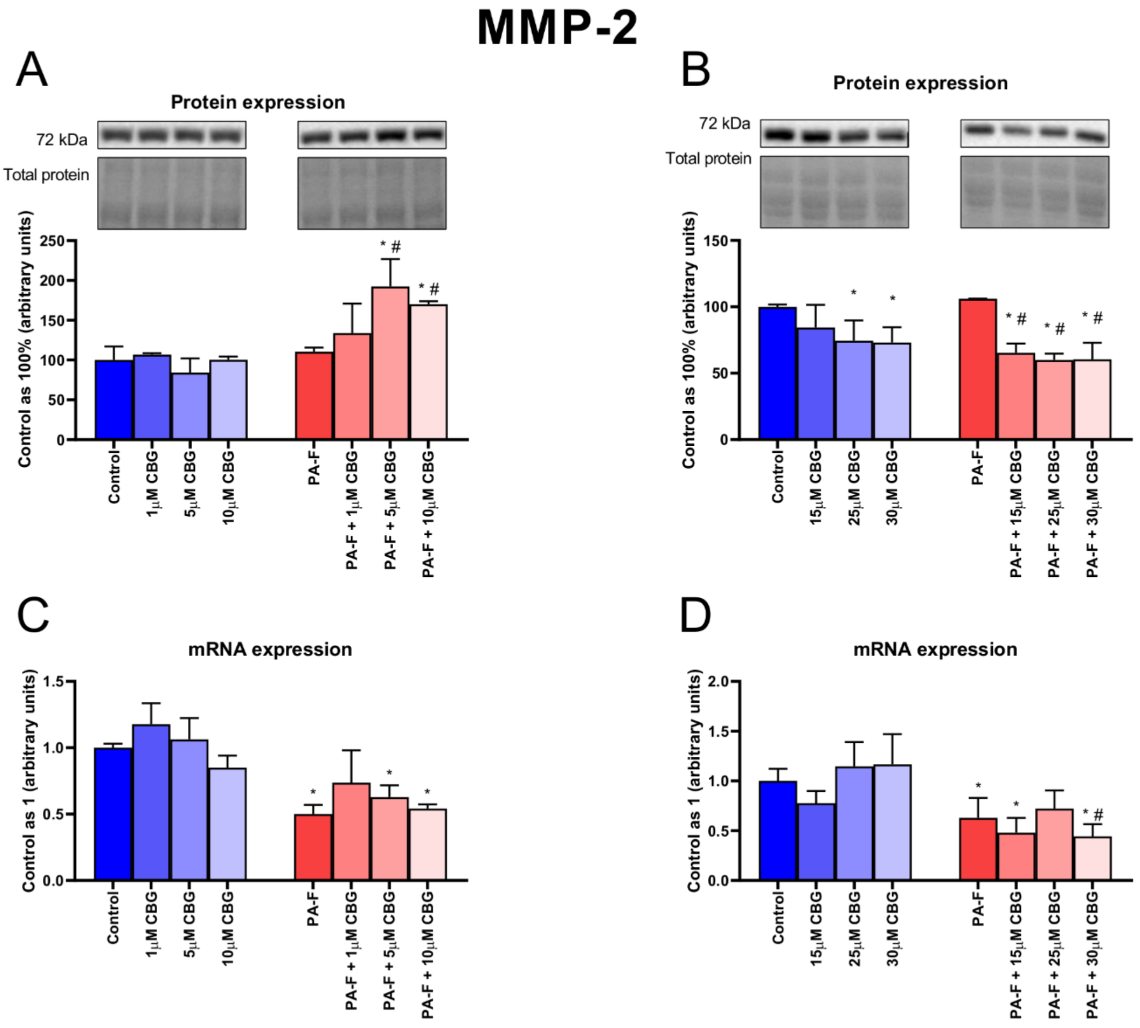

Figure S2. The influence of cannabigerol (CBG), in a concentration-dependent manner, on the matrix metalloproteinase 2 (MMP-2) protein expressions (A, B), mRNA expressions (C, D) in hepatocytes exposed to standard media (Control) or palmitate and fructose media (PA-F). The cells were treated with different concentrations of CBG for 48h. The MMP-2 protein and mRNA expression assays were performed by the Western blot and RT-PCR techniques, respectively. The data were expressed as mean ± SD of six independent determinations and presented as a percentage of MMP-2 changes relative to the Control (set as 100% for Western blot and as 1 for RT-PCR). The significant differences were indicated in comparison with the Control group (*$p<0.05$) and PA-F group ($^{\#}p<0.05$).

1.3. Concentration-dependent influence of cannabigerol on matrix metalloproteinase 9 changes in post-incubation media and primary rat hepatocytes exposed to palmitate in combination with fructose

The hepatocytes treatment with 1μM CBG, 5μM CBG and 10μM CBG in combination with the palmitate and fructose caused a decline in the MMP-9 protein expression (PA-F + 1μM CBG: -18.0% and -18.1%, PA-F + 5μM CBG: -26.3% and -26.3%, PA-F + 10μM CBG: -20.2% and -20.2%, $p<0.05$, Figure S3A) compared to the Control and PA-F groups, respectively. The MMP-9 protein expression was raised in the PA-F + 25μM CBG group (+11.1%, $p<0.05$, Figure S3B, vs. Control group).

The mRNA expression of MMP-9 was decreased in both PA-F groups (-42.9% and -48.3%, $p<0.05$, Figure S3C and S3D, respectively). The mRNA expression of MMP-2 was also significantly reduced in selected examined groups (10μM CBG: -28.6%, PA-F + 5μM CBG: -3.6%, 30μM CBG: -34.0%, PA-F +

15µM CBG: -54.7%, PA-F + 25µM CBG: -55.5%, PA-F + 30µM CBG: -63.2%, $p<0.05$, Figure S3C and S3D) compared to the proper Control group. We noticed an increment in the MMP-9 mRNA expression in hepatocytes treated with 10µM CBG in the PA-F condition (+68.8%, $p<0.05$, Figure 8C, vs. PA-F group). Our study also demonstrated a lower mRNA expression of MMP-9 after high concentrations of CBG treatment of cells exposed to palmitate and fructose media (PA-F + 15µM CBG: -12.5%, PA-F + 25µM CBG: -14.0%, PA-F + 30µM CBG: -28.8%, $p<0.05$, Figure S3D) in comparison with the PA-F group.

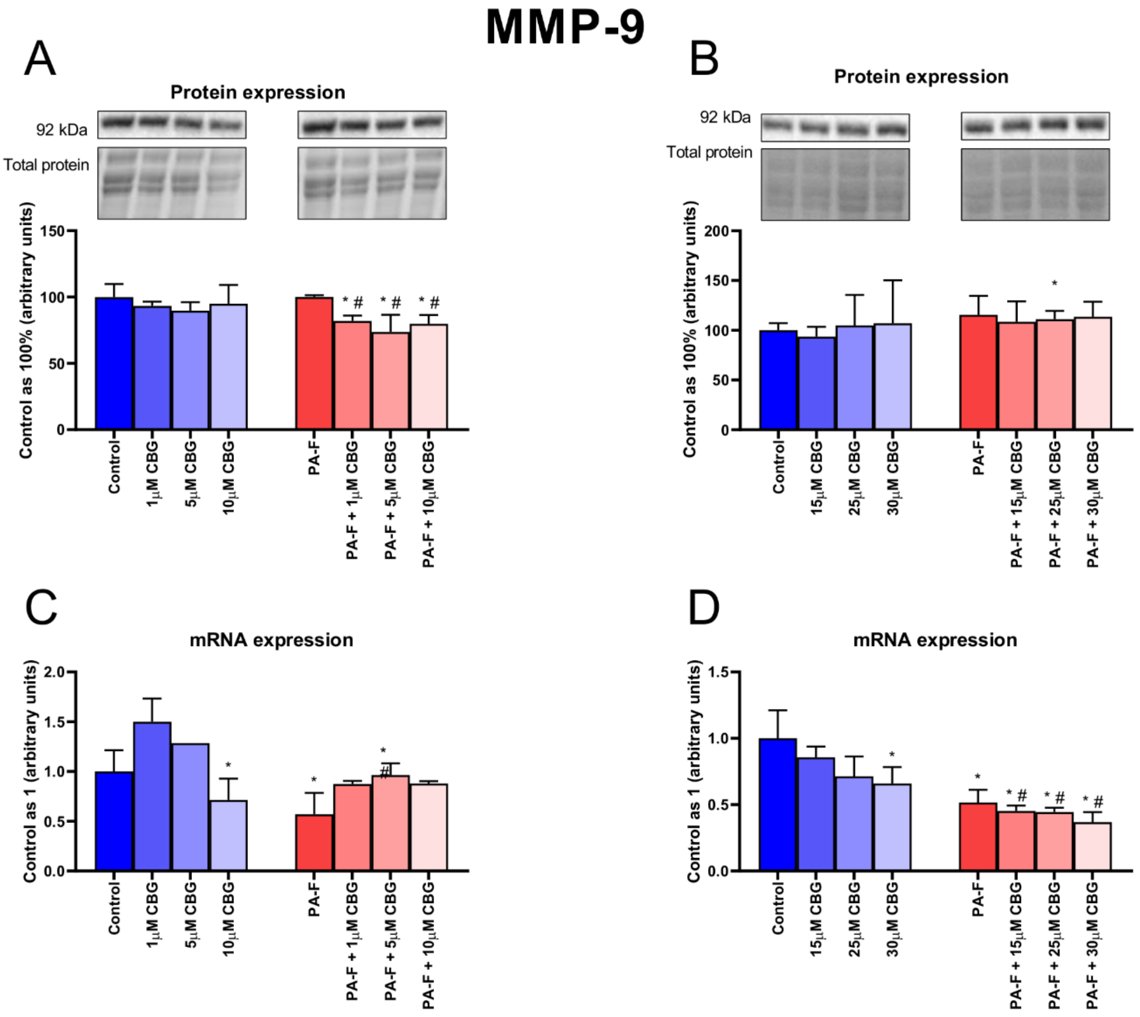

Figure S3. The influence of cannabigerol (CBG), in a concentration-dependent manner, on the matrix metalloproteinase 9 (MMP-9) protein expressions (A, B), mRNA expressions (C, D) in hepatocytes exposed to standard media (Control) or palmitate and fructose media (PA-F). The cells were treated with different concentrations of CBG for 48h. The MMP-9 protein and mRNA expression assays were performed by the Western blot and RT-PCR techniques, respectively. The data were expressed as mean ± SD of six independent determinations and presented as a percentage of MMP-9 changes relative to the Control (set as 100% for Western blot and as 1 for RT-PCR). The significant differences were indicated in comparison with the Control group (*$p<0.05$) and PA-F group ($^{\#}p<0.05$).

1.4. Concentration-dependent influence of cannabigerol on tissue inhibitor of metalloproteinase 1 changes in post-incubation media and primary rat hepatocytes exposed to palmitate in combination with fructose

The protein expression of TIMP-1 was changed only in two examined groups, i.e., PA + 15µM CBG and PA-F + 25µM CBG. There was an increment in hepatocytes incubated with 15µM CBG (+140.7% and +108.8%, $p<0.05$, Figure S4B, vs. Control and PA-F groups, respectively) and a reduction in hepatocytes incubated with 25µM CBG (-20.7%, $p<0.05$, Figure S4B, vs. Control group).

The mRNA expression of TIMP-1 was increased in both PA-F groups (+57.3% and +58.3%, $p<0.05$, Figure S4C and S4D, respectively). The TIMP-1 mRNA expression was reduced after cells treatment

with low concentrations of CBG in the combination of palmitate and fructose (PA-F + 1µM CBG: -49.4%, PA-F + 5µM CBG: -53.9%, PA-F + 10µM CBG: -33.6%, $p<0.05$, Figure S4C) than in the PA-F group. Moreover, in relation with the Control group, the TIMP-1 mRNA expression was declined after hepatocytes incubation with PA-F and 5µM CBG (-27.5%, $p<0.05$, Figure S4C).

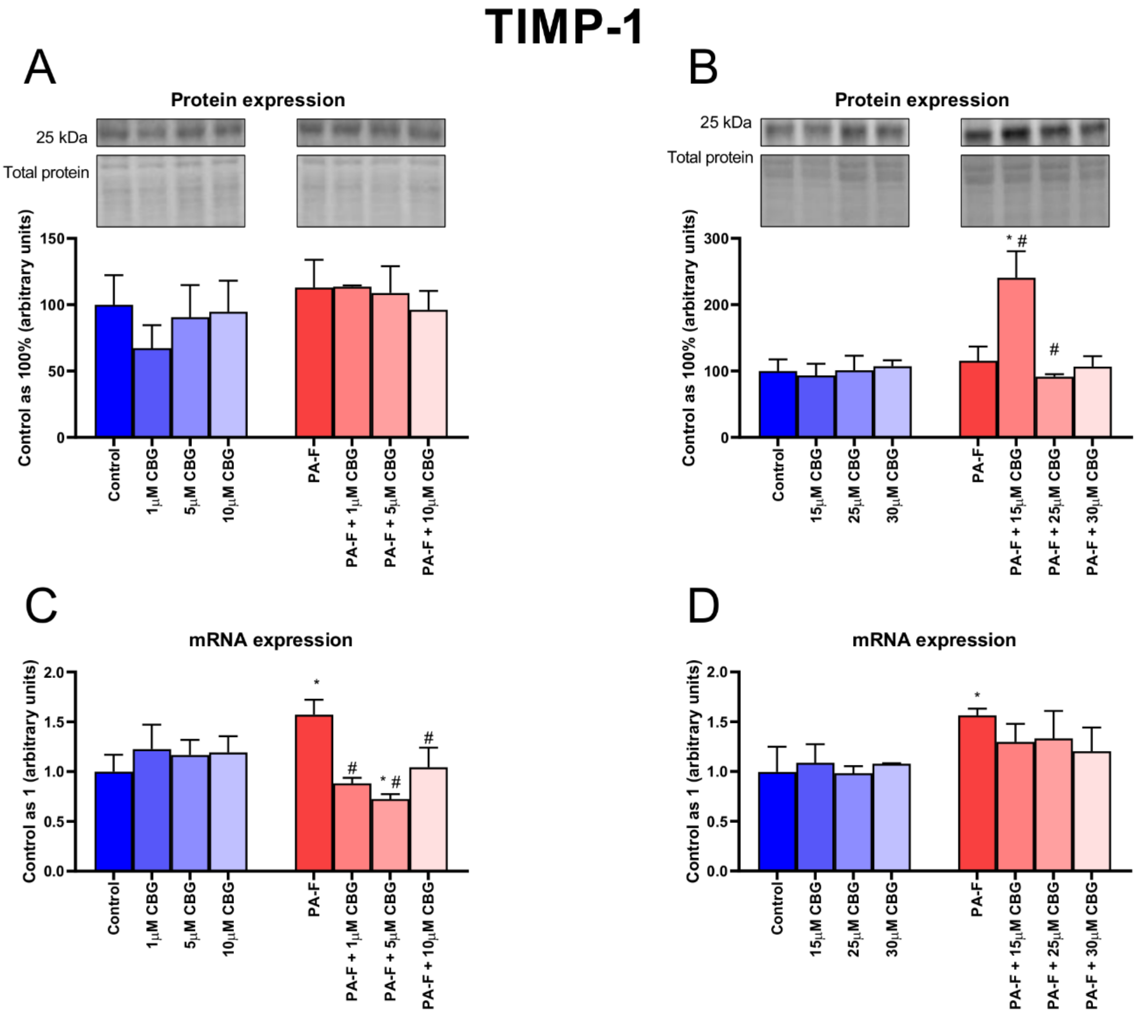

Figure S4. The influence of cannabigerol (CBG), in a concentration-dependent manner, on the tissue inhibitor of metalloproteinase 1 (TIMP-1) protein expressions (A, B), mRNA expressions (C, D) in hepatocytes exposed to standard media (Control) or palmitate and fructose media (PA-F). The cells were treated with different concentrations of CBG for 48h. The TIMP-1 protein and mRNA expression assays were performed by the Western blot and RT-PCR techniques, respectively. The data were expressed as mean ± SD of six independent determinations and presented as a percentage of TIMP-1 changes relative to the Control (set as 100% for Western blot and as 1 for RT-PCR). The significant differences were indicated in comparison with the Control group (*$p<0.05$) and PA-F group ($^{\#}p<0.05$).

1.5. Concentration-dependent influence of cannabigerol on tissue inhibitor of metalloproteinase 2 changes in post-incubation media and primary rat hepatocytes exposed to palmitate in combination with fructose

In comparison with proper Control group the protein expression of TIMP-2 was decreased in the 5µM CBG, 10µM CBG, PA-F + 1µM CBG, PA + 5µM CBG, and PA + 5µM CBG groups (-29.4%, -28.5%, -31.3%, -23.1%, and -20.3%, $p<0.05$, Figure S5A and S5B, respectively) and increased in the PA-F + 10µM CBG and PA-F + 30µM CBG groups (+16.5% and +27.9%, $p<0.05$, Figure S5A and S5B, respectively). We also observed a diminishment in the TIMP-2 protein expression after treatment with 1µM CBG, 5µM CBG, 15µM CBG, and 25µM CBG in the PA-F condition (-40.7%, -33.6%, -32.4%, -8.9%, $p<0.05$, Figure S5A and S5B) than in the PA-F group.

The mRNA expression of TIMP-2 was decreased in the PA-F + 1µM CBG, PA-F + 5µM CBG, PA-F + 15µM CBG, and PA-F + 25µM CBG groups (-20.4%, -28.1%, -20.4% and -12.8%, $p<0.05$, Figure S5C and S5D, respectively) in comparison with the proper Control group. In relation to the PA-F group, the TIMP-2 mRNA expression was attenuated by cannabigerol treatment with the following groups: PA-F + 1µM CBG, PA-F + 5µM CBG, and PA-F + 15µM CBG (-21.9%, -29.4%, and -19.3%, $p<0.05$, Figure S5C and S5D, respectively).

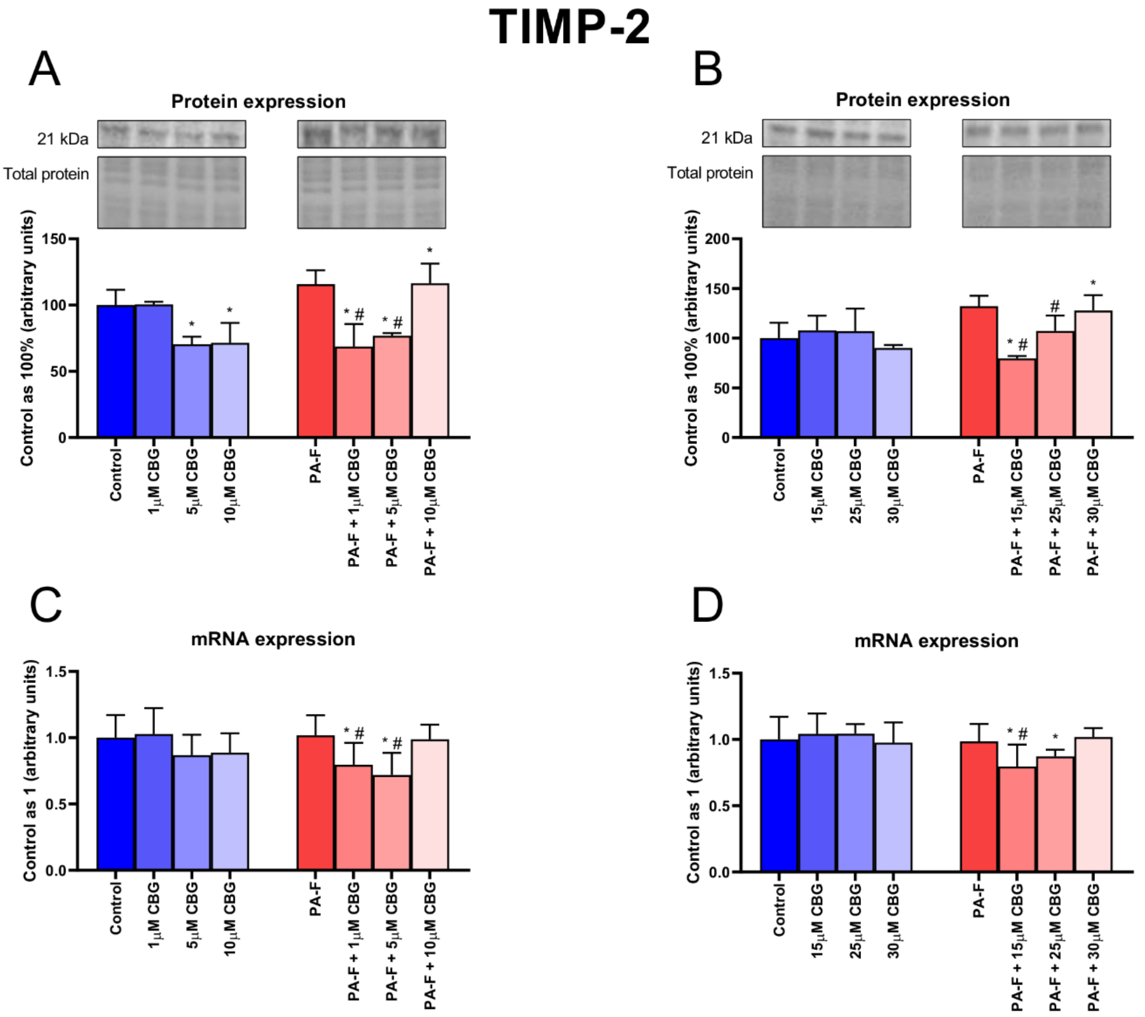

Figure S5. The influence of cannabigerol (CBG), in a concentration-dependent manner, on the tissue inhibitor of metalloproteinase 2 (TIMP-2) protein expressions (A, B), mRNA expressions (C, D) in hepatocytes exposed to standard media (Control) or palmitate and fructose media (PA-F). The cells were treated with different concentrations of CBG for 48h. The TIMP-2 protein and mRNA expression assays were performed by the Western blot and RT-PCR techniques, respectively. The data were expressed as mean ± SD of six independent determinations and presented as a percentage of TIMP-2 changes relative to the Control (set as 100% for Western blot and as 1 for RT-PCR). The significant differences were indicated in comparison with the Control group ($^*p<0.05$) and PA-F group ($^\#p<0.05$).
